# Acute abdominal pain in women of reproductive age: keys to suggest a complication of endometriosis

**DOI:** 10.1186/s13244-023-01433-6

**Published:** 2023-05-24

**Authors:** Juliette Coutureau, Caroline Mandoul, Cecile Verheyden, Ingrid Millet, Patrice Taourel

**Affiliations:** Department of Medical Imaging, CHU Lapeyronie, Universitary Hospital of Montpellier, 371 Avenue du Doyen Gaston Giraud, 34295 Montpellier, France

**Keywords:** Endometriosis complication, Emergency, Acute abdominal pain, Imaging

## Abstract

Although endometriosis is a common gynecological condition in women of reproductive age, a complication of endometriosis is rarely considered as the differential diagnosis of acute abdominal pain in that context. However, acute events in women with endometriosis can represent life-threatening conditions, which require emergent treatment and often surgical management. Mass effect of endometriotic implants can give rise to obstructive complications, specifically occurring in the bowel or in the urinary tract, while inflammatory mediators released by ectopic endometrial tissue can lead to inflammation of the surrounding tissues or to superinfection of the endometriotic implants. Magnetic resonance imaging is the best imaging modality to reach the diagnosis of endometriosis, but an accurate diagnosis is possible on computed tomography, especially in the presence of stellar, mildly enhanced, infiltrative lesions in suggestive areas. The aim of this pictorial review is to provide an image-based overview of key findings for the diagnosis of acute abdominal complications of endometriosis.

**Graphical Abstract:**

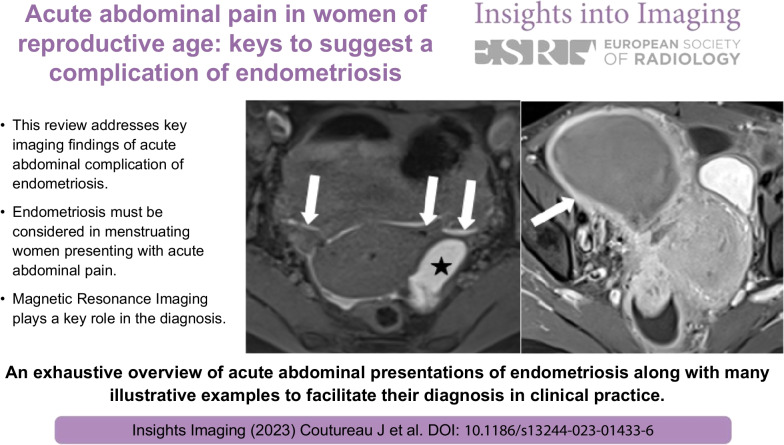

## Critical relevance statement

This educational review provides an exhaustive overview of acute abdominal presentations of endometriosis along with illustrative examples, to facilitate their diagnosis in clinical practice.

## Key points

- This review addresses key imaging findings of acute abdominal complication of endometriosis.
- Endometriosis must be considered in menstruating women presenting with acute abdominal pain.
- Magnetic Resonance Imaging plays a key role in the diagnosis.

## Introduction

Endometriosis is characterized by the presence of endometrial tissue in sites other than the uterine cavity [1, 2]. Ectopic implants are mostly located in the pelvis, although they can be found in multiple abdominal and thoracic sites [3, 4]. Endometriosis is a common gynecological condition affecting women in their reproductive years. Its prevalence is of approximately 10% in women of reproductive age, 20–50% in women with infertility, and up to 90% in women with chronic pelvic pain [5, 6]. Since it is an estrogen-dependent disorder, ectopic endometrial tissue cyclically undergoes decidualization, which leads to inflammation and fibrosis [7, 8]. Both a mass effect and an inflammatory cascade are responsible for the clinical manifestations of endometriosis and may lead to acute complications. First, the mass effect gives rise either to compression of a nerve with acute pain or to obstructive complications occurring in the bowel or in the urinary tract [9–11]. Second, endometriotic ectopic tissue can release inflammatory mediators, such as growth factors and cytokines, which lead to inflammation of the surrounding tissues and to an inflammatory disease [2].

Complicated endometriosis is rarely considered as the differential diagnosis of acute abdominal pain in women of reproductive age. Likewise, acute abdominal pain in patients with endometriosis may be hastily dismissed as usual pelvic pain, without being considered as a complication of endometriosis [7, 12, 13]. The aim of this pictorial review is to analyze imaging findings of acute abdominal complications of endometriosis, and to provide tools for the radiologists in their daily practice to help them make an accurate diagnosis.

## Gynecologic complications

### Pelvic inflammatory disease

**Fig. 1:**
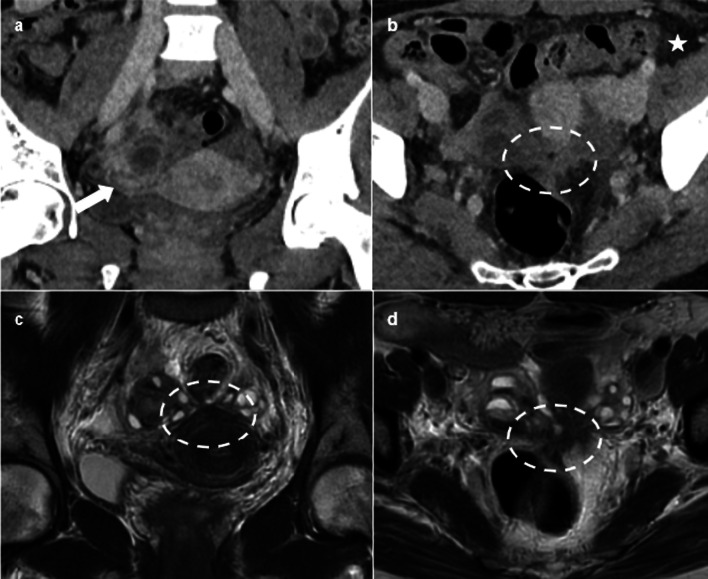
44-year-old woman presenting with pelvic pain and fever. CT (**a**, **b**) depicts signs of acute salpingitis, associating a strongly enhancing right fallopian tube (arrow) and pelvic fat stranding (star). On axial CT (**b**), the location of the ovaries is suggestive of deep endometriosis. T2W MRI (**c**, **d**) confirms the presence of an endometriotic nodule of the torus, responsible for a “kissing of the ovaries” (dotted circle)

Pelvic inflammatory disease (PID) in women with endometriosis is more common, more severe, often refractory to antibiotic treatment and requiring surgical intervention [14, 15]. Most of the cases of PID in women with endometriosis occur after assisted reproductive techniques (ART), generally within the year after ART [7, 16]. Imaging of PID in patients with endometriosis shows findings of severe PID, including acute salpingitis, oophoritis, pyosalpinx, hydrosalpinx, tubo-ovarian abscess (Fig. 1). In this context of PID, the presence of a thickening of the uterosacral ligaments is not particularly suggestive of endometriosis.

### Endometrioma superinfection

Superinfection approximately complicates 2% of endometriomas [17]. The propensity of endometriomas to become infected has been attributed to locally reduced resistance to infection, as well as to endometriotic blood products acting as an effective culture medium [14, 18, 19]. The mechanisms leading to infection of endometriomas are multiple: ascending route in a context of lower genital tract infection, hematogenous spread, extension from adjacent bowel, or direct inoculation after a surgical procedure or transvaginal aspiration [20]. In a tertiary university-based reference center for endometriosis, 30% of endometrioma superinfections occurred after oocyte retrieval [21]. Spontaneous rupture of ovarian endometriotic cysts has also been shown to be significantly associated with endometrioma superinfection [20].

The clinical presentation overlaps with that of other abdominal and pelvic infections, the patient presenting with progressive lower abdominal pain with fever and elevated C-reactive protein (CRP) with leukocytosis [22]. However, superinfection of an endometrioma should be considered in women of reproductive age, especially with a background history of pelvic endometriosis, when an inflammatory syndrome is present.

**Fig. 2:**
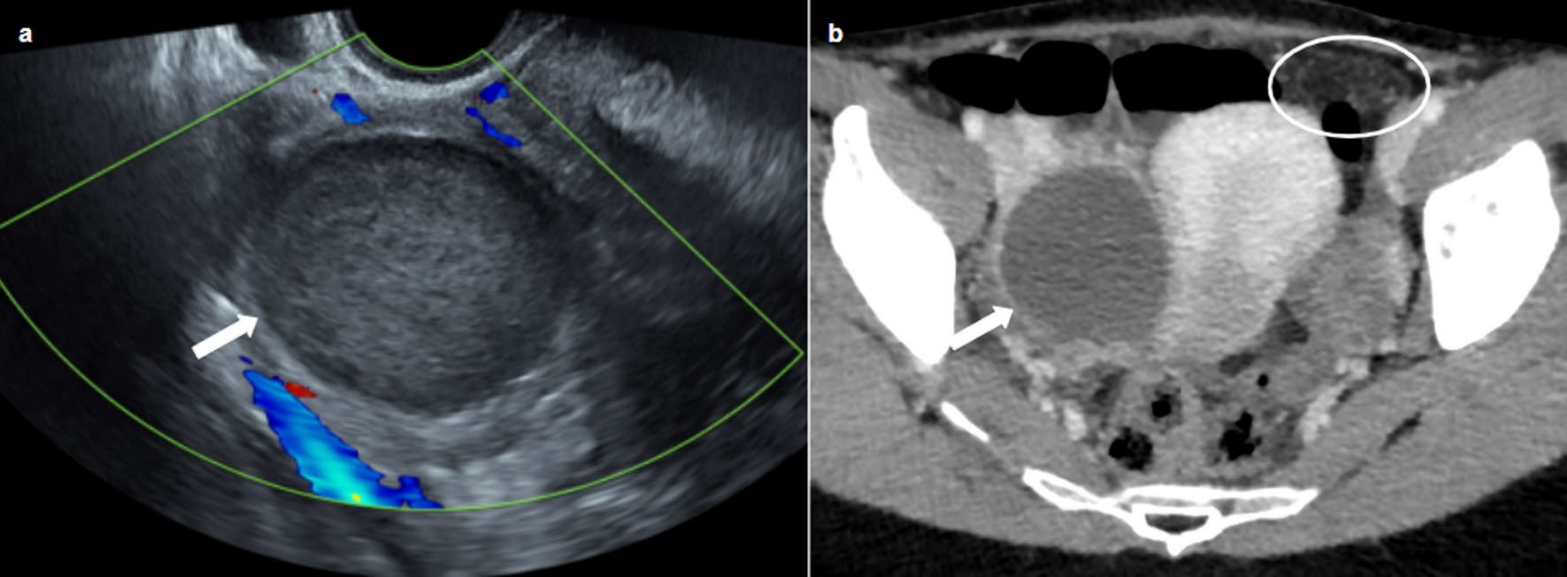
35-year-old female presenting with pelvic pain and elevated C-reactive protein (CRP). Transvaginal ultrasonography (**a**) showing a typical endometrioma of the right ovary: unilocular cyst with diffuse homogeneous ground-glass echoes. CT (**b**) showing a nonspecific cystic mass with uniformly low attenuation content (arrow), associated with pelvic fat stranding (circle)

**Fig. 3:**
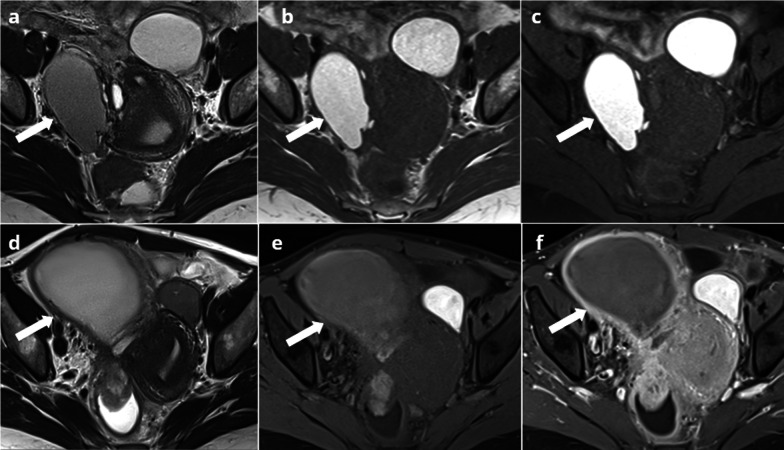
39-year-old female with deep endometriosis. Typical endometrioma of the right ovary (arrow): unilocular cyst with shading on T2W images (**a**) hypersignal > fat on T1W images (**b**), persisting on T1W FS images (**c**). Several months later, she presents with pelvic pain, fever and hyperleukocytosis. MRI shows an increase in size of the right endometrioma (arrow), with loss of shading on T2W (**d**), loss of hyperintensity on T1W FS (**e**) and a thick, enhanced wall on T1W FS after contrast injection (**f**)

Sonographic appearance may be similar to that of uninfected endometrioma, but the thickening of the endometrioma wall associated with flow on Doppler may suggest the diagnosis. CT usually shows a nonspecific cystic mass with uniformly low attenuation content [23] (Fig. 2). MRI is the key to diagnosis, associating: an increase in size of a known endometrioma, a loss of typical endometriotic signal (loss of hyperintensity in T1 weighted fat sat and loss of shading in T2), a marked restriction of diffusion with very low ADC values, thick enhanced walls of the endometrioma and pelvic fat stranding [24] (Fig. 3).

There is no established standard of care for infected endometriomas [25]. Antibiotics are usually ineffective, and percutaneous CT-guided or US-guided drainage might lead to intraperitoneal spillage of cyst contents, which could trigger an inflammatory response leading to worsened pelvic pain, adhesions and/or infertility. Therefore, laparoscopic cystectomy is often performed [19, 25].

### Endometrioma rupture

Rupture of an endometrial cyst is a relatively rare, but not exceptional, condition. In a 7-year retrospective study including 720 women with surgery of endometriotic cyst, the prevalence of rupture was 2.2% [26]. Besides, in a large series of gynecological acute abdomen, 4.6% (70/1509) were attributed to rupture of an endometrial cyst [27].

This complication most commonly occurs during pregnancy, because of the rapid growth of endometrioma due to hormonal stimulation [28, 29]. Clinically, endometrioma rupture may mimic the rupture of a hemorrhagic ovarian cyst with findings ranging from symptoms of inflammation, such as fever, leukocytosis, and elevated c-reactive protein, to acute abdominal pain and signs of hypovolemic shock [30, 31].

**Fig. 4:**
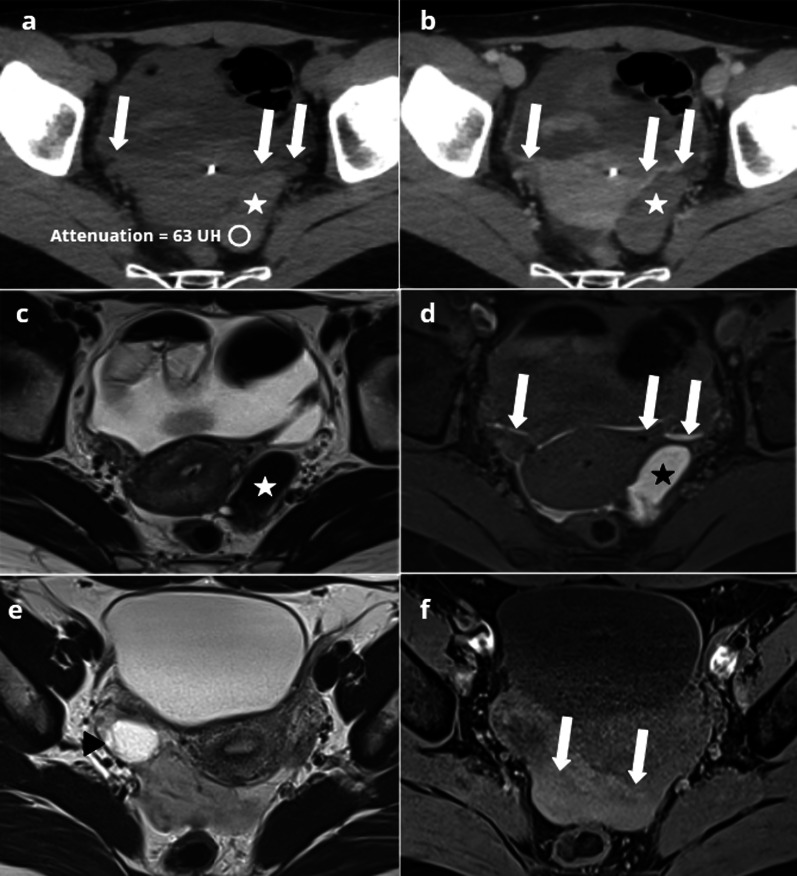
42-year-old female presenting with a non-febrile acute pelvic pain, with negative human chorionic gonadotropin (HCG) and C-reactive protein (CRP). Non-enhanced CT (**a**) showing a spontaneously dense left ovarian mass (star) with free intraperitoneal fluid (arrows). After contrast injection (**b**), no enhancement of the mass is observed. MRI in the same patient (**c**, **d**) showing a distorted left ovarian mass (star) in low signal on T2W images **(c)** and high signal in T1W images (**d**). Free intraperitoneal fluid (arrows) presents a hemorrhagic level showing the same signal as the endometrioma (**d**). 27-year-old female patient presenting with right corpus luteal cyst rupture. MRI (**e**, **f**) shows a heterogeneous cyst (arrowhead) on T2W images (**e**), and free intraperitoneal fluid (arrows) showing an intermediate signal on T1W images (**f**)

US shows classical findings of an endometrioma with ascites, the association of these two features being highly suggestive of a rupture [32]. CT may show features similar to those of corpus luteal cyst rupture, save that intraperitoneal fluid could be more homogeneous and of lower density [33, 34] (Fig. 4).

MRI only is specific to the diagnosis, especially when endometriosis is unknown. MRI usually shows a distorted endometrioma, associated with high signal ascites on T1-weighted images, equal to or slightly lower than that of the endometrial cyst. By contrast, in corpus luteal cyst rupture, the hemoperitoneum is more likely to appear in intermediate signal on T1-weighted sequence, with a sentinel clot in high signal (Fig. 4) [35–37].

Clinical and imaging findings allowing to differentiate the two entities are presented in Table 1.

**Table 1:** Clinical and Imaging findings to differentiate ruptured endometrioma from ruptured corpus luteal cyst

	Ruptured endometrioma	Ruptured corpus luteal cyst
Clinical and biological findings	Fever and leukocytosis common	Fever and leukocytosis rare
Cyst size	4–8 cm	1–4 cm
Cyst signal	Homogeneous hyperintense on T1 sequence Shading sign	Heterogeneous on T1 and T2 sequences No shading sign T2 dark spot sign Enhanced rim
Peritoneal fluid signal	High signal on T1W sequences, equal to that of the endometrioma	Low or intermediate signal on T1W sequences

### Endometrioma torsion

Endometrioma torsion is exceptional, since endometriomas are often firmly adherent to adjacent structures. In a retrospective study including 225 patients operated for endometrioma, 22.7% of which underwent emergent surgery, no cases of torsion were reported [38]. Rather, few case reports to date depict isolated fallopian tube torsion in women with endometriosis, occurring on pre-existing hematosalpinx, which results from the obstruction of the fimbrial end of the tube and of retrograde menstruation.

## Digestive complications

Bowel endometriosis, which occurs in 5–12% of women affected by endometriosis, more frequently involves the rectosigmoid, responsible for about 90% of all digestive lesions, followed by the ileum, the appendix and the caecum. In a prospective observational series of 1101 patients with endometriotic lesions seen at laparoscopy, 8.4% had involvement of the sigmoid colon and less than 0.5% had involvement of the caecum or of the appendix [39].

Symptoms associated with deep infiltration of the bowel include periodic abdominal pain, dysmenorrhea, constipation, diarrhea, pain during defecation, and periodic rectal bleeding [40–43]. These complaints are due to extensive fibrosis caused by chronic inflammation of the endometriosis, but a distinct acute worsening of symptoms is possible, although rare, in this disease [44].

The diagnosis of endometriosis complication is rarely made on imaging but may be suggested in a context of known endometriosis with digestive involvement [7, 45].

### Bowel obstruction

Bowel obstruction is a rare event in endometriosis, complicating about 5‰ of endometriosis [46, 47]. The pathophysiology of obstruction in endometriosis is multi-faceted: adhesions related to endometriosis itself or to abdominal surgery for endometriosis or, more rarely, intestinal intussusception [7, 48, 49]. The diagnosis must be considered in a patient with clinical findings of bowel obstruction, if there is a history of endometriosis, or in a woman of childbearing age [50]. Some ancillary findings may also be helpful: a prolonged history of relapsing symptoms of bowel obstruction, or a history of infertility [51].

**Fig. 5:**
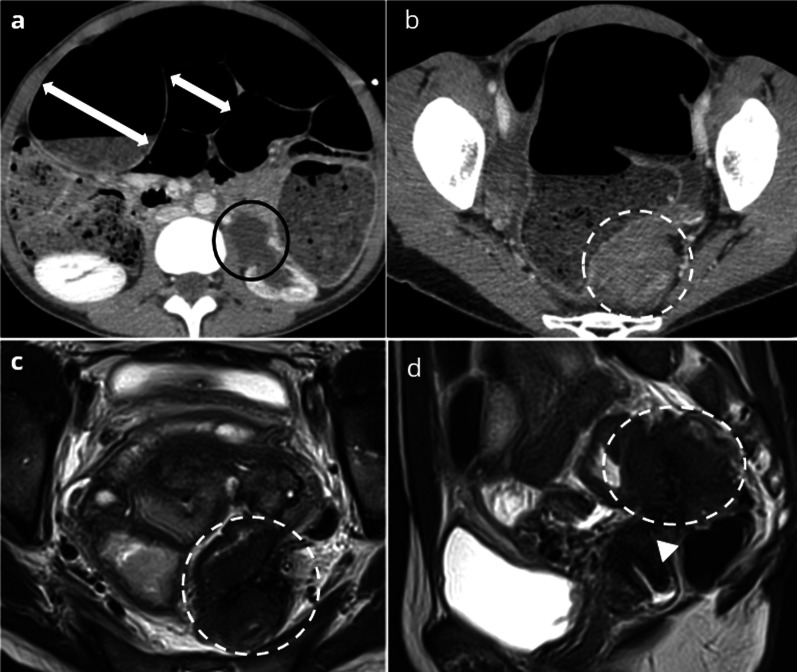
39-year-old female presenting with acute clinical intestinal obstruction. Axial enhanced CT (**a**, **b**) shows a large and small bowel distension (arrows), as a consequence of obstruction by a recto-sigmoid mass (white dotted circle, (**b**)). Features of long-standing left urinary obstruction (black plain circle) may be suggestive of deep endometriosis (**a**). T2W MRI (**c**, **d**) shows a stellar retractile hypoT2 mass of the Douglas pouch with colic asymmetric muscular thickening (white dotted circle) consistent with deep endometriosis of the torus (arrowhead) extending transmurally to the recto-sigmoid with stenosis and left ureter involvement

In a patient with an acute abdomen, CT is considered as the reference imaging examination. Features depend on the site and on the mechanism of the obstruction. Large bowel obstruction is the most common site of bowel obstruction in endometriosis, the sigmoid colon and/or the rectum being involved in 2/3 of cases [7]. CT shows a soft-tissue mass at the transition point, with mild to moderate contrast enhancement, responsible for an eccentric and non-circumferential thickening of the bowel wall [52–54]. MRI is more specific than CT, showing a retractile hypointense pelvic mass in T2-weighted sequences, invading the rectum or colon muscular layer, with a classical “mushroom cap sign” as the mass protrudes into the intestinal lumen. T1-weighted sequences with gadolinium can be helpful to confirm the sparing of the mucosal layer [55–57] (Fig. 5). US has the same performance as MRI for the diagnosis of rectosigmoid endometriosis, showing a hypoechoic thickening of the anterior wall of the rectosigmoid colon [58].

The main differential diagnosis is bowel cancer [7, 54], but the asymmetrical pattern of the stenosis is suggestive of endometriosis, since the lesion initially involves the serosa, infiltrates the muscular layer, but only rarely invades the mucosa, whereas colon carcinoma is an intrinsic lesion starting in the mucosa [6]. DWI MRI may also help differentiate endometriosis from colon carcinoma, since carcinoma shows high signal intensity on DWI images due to high cellularity, whereas endometriosis shows low signal intensity due to the T2-blackout effect [59]. Sigmoidoscopy, if performed, would confirm the extrinsic compression of the colon without involvement of the mucosa [60, 61].

In small bowel obstruction, CT may show a fibrous mass attached to the serosa or, more frequently, classical findings of adhesions [45], with the presence of a beak sign and the absence either of a mass at the transition zone, or of a bowel wall thickening.

Intussusception is a rare cause of bowel obstruction and an exceptional complication of endometriosis with a few case reports of appendiceal and ileal intussusception [46, 49]. To our knowledge, the physiopathology of intussusception in endometriosis is not well known. CT shows classic features of intussusception, including the target or sausage sign and the presence of mesentery in the digestive lumen [62]. The endometriotic cause of the intussusception is generally not evoked on CT, and a lead point is not identified [63]. Therefore, while searching for a tumor (present in 1/3 of ileal invaginations and in 2/3 of colonic invaginations) is the rule [64], endometriosis should be considered in women of reproductive age, even though the association between endometriosis and intussusception is rare [49].

Identifying the cause of bowel obstruction is of importance in patients with endometriosis, because extrinsic masses responsible for bowel obstruction need bowel resection, adhesions are often medically treated, and ileocolic intussusception due to endometriosis preferably requires a surgical approach and responsible for bowel obstruction [51].

### Bowel perforation

**Fig. 6:**
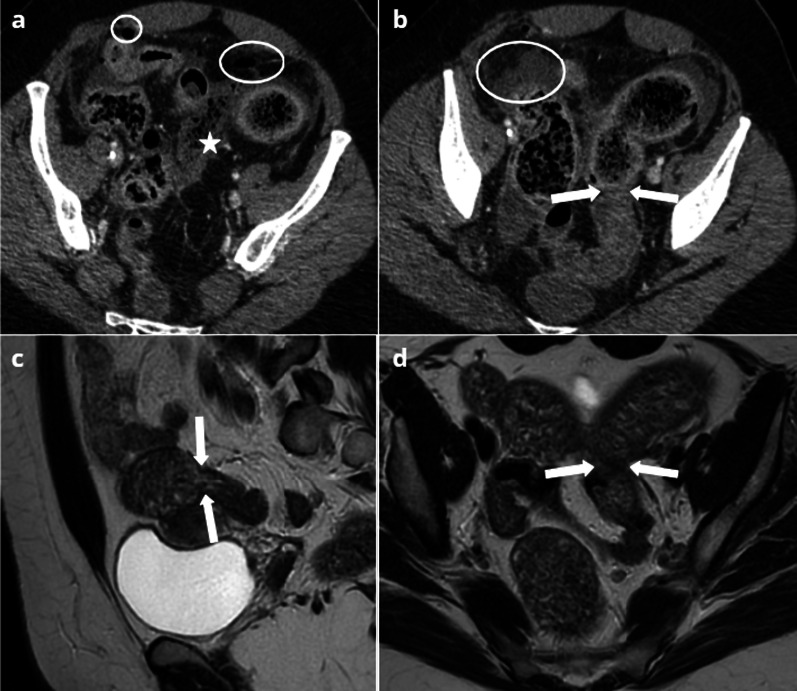
39-year-old female presenting with acute abdominal pain and abdominal tenderness at physical examination. Axial enhanced CT (**a**, **b**) shows findings of acute peritonitis: pneumoperitoneum (circles), peritoneal effusion (circles), peritoneal thickening, and fecal material within the peritoneal cavity (star). The presence of fecal material close to the sigmoid colon is suggestive of the site of perforation (**a**). A stenosis of the sigmoid is suspected (arrows, (**b**)). The patient had a history of shaving surgery for rectosigmoid endometriosis and had a pelvic MRI a few weeks before. T2W MRI (**c**, **d**) confirms the presence of a fibrotic stenosis of the sigmoid (arrows), most probably secondary to shaving surgery

Perforation of the bowel wall is a very rare outcome of endometriosis with less than 30 cases reported in the literature, including small bowel, large bowel and appendix perforations [45], most of them occurring during pregnancy. The pathophysiology of bowel perforation secondary to intestinal endometriosis is uncertain: weakness of the intestinal wall affected by foci of endometriosis, ischemic or inflammatory events secondary to endometriosis, or postoperative damage to the digestive wall [45]. The rarity of perforation is related to that of mucosal involvement in digestive endometriosis. A recent meta-analysis reported a 1.7% rate of bowel perforation during surgery and a 1.7–2.2% rate of late bowel perforation after the shaving technique [65]. CT is recommended in case of suspicion of digestive perforation. It shows the usual features of perforation: free intraperitoneal gas and fluid, or intra-abdominal collections with ectopic gas (Fig. 6).

### Acute appendicitis

The estimated incidence of appendiceal endometriosis is less than 1% in patients with deep endometriosis [39]. Besides, in the general population, involvement by endometriosis accounts for less than 1% of appendectomy specimens [66]. Endometriotic lesions preferentially affect the body and the tip of the appendix [3]. Clinical presentation is variable. Endometriosis of the appendix is generally asymptomatic and discovered on pathologic evaluation after appendectomy. When symptomatic, patients can have right lower quadrant pain typical for appendicitis and rarely suggestive of endometriosis, unless cyclical or worsening during menses [67]. Exceptionally, appendiceal endometriosis can be revealed by a complication, such as lower gastrointestinal bleeding, intestinal perforation or intestinal obstruction from intussusception [68].

Acute presentation of appendicular endometriosis cannot be clinically distinguished from acute appendicitis. US or CT may then show three different patterns [68]:

- usual imaging findings of acute appendicitis;
- imaging findings consistent with an appendicular mucocele with an isolated non-specific dilatation of the appendix;
- hypoechoic and/or mildly enhanced soft-tissue mass involving the appendix, which is the most suggestive, albeit often very subtle [31], feature of the disease.

**Fig. 7:**
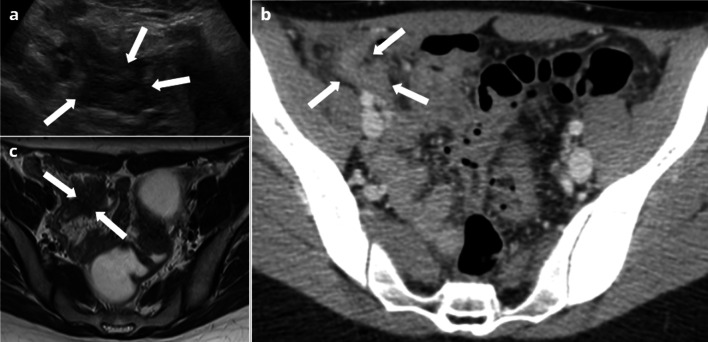
26-year-old female presenting with acute right lower quadrant pain. US and CT (**a**, **b**) show an irregular pseudonodular hypoechoic (**a**) enhanced (**b**) thickening of the appendix (arrows), with pelvic fat stranding. T2W MRI (**c**) shows a fibrotic endometriotic implant of the right ovarian fossa, invading the appendix (arrows). Surgery was performed and found hemoperitoneum along with many endometriotic implants of the ileocecal region. Pathological reports found endometrial stroma in the muscular layer of the appendix

MRI rarely suggests endometriosis as the cause of acute appendicitis [7, 69]. However, if performed, T2-weighted sequences depict a hypointense retractile mass or nodular thickening of the appendix. Involvement of the appendix can also be contiguous to fibrotic implants of the ovarian fossa [70, 71] (Fig. 7).

Although endometriosis is the most likely diagnosis in patients with a known deeply infiltrating endometriosis and mass within the appendix, carcinoid tumors are the most common neoplasm of the appendix in young patients and should be included in the differential diagnosis [72].

In clinical practice, when appendiceal endometriosis is isolated without pelvic implants, the diagnosis is not performed by imaging [7, 71]. Conversely, the diagnosis may be evoked when features of deep endometriosis coexist with appendiceal findings on imaging. Yet, evoking endometriotic appendicitis before surgery is of importance, as it may require a multidisciplinary team approach [66].

## Peritoneal complications

### Inflammation

The deposits of endometriotic implants on the peritoneum are classically superficial, measuring less than 5 mm, and defining superficial peritoneal endometriosis. In some cases, inflammatory processes within the peritoneal cavity may complicate endometriosis. Peritoneal inflammation may then lead to abdominal pain just like other endometriotic lesions, due to distortion of the pelvic anatomy, extensive adhesions, cyclical bleeding, neural and perineural invasion [12].

**Fig. 8:**
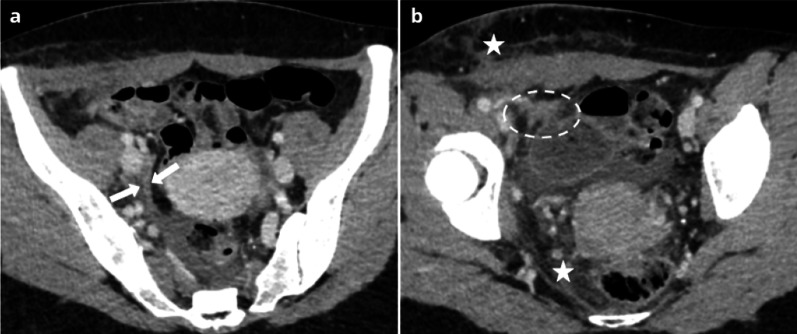
27-year-old female presenting with acute right lower quadrant pain and elevated CRP. CT (**a**, **b**) shows a normal appendix (arrows), but subcutaneous and pelvic fat stranding (star), along with a stellar retractile mass of the right iliac fossa (dotted circle). Surgical exploration found no sign of appendicitis or of right adnexal abnormality, but it revealed inflammatory peritoneal endometriotic implants of the right lower quadrant. A good clinical outcome was achieved with hormonal treatment

Superficial lesions are usually difficult to detect by imaging, although hemorrhagic implants can be seen on MRI as hyper T1 foci [73]. CT findings may vary from isolated peritoneal fat stranding or located stellar peritoneal mass (Fig. 8) to marked inflammation of the peritoneum with the mesos mimicking peritoneal carcinomatosis [74].

Implants are well recognized at laparoscopy: they appear as black, white, or red spots, depending on the degree of fibrosis, scarring, and hemorrhage within the lesion.

Endometriosis-related encapsulated peritonitis has also been described a few times in the literature [75, 76]. It has been hypothesized that the peritoneal irritation caused by endometriosis resulted in extensive fibrosis and inflammation. CT depicts massive ascites, centrally encased bowel and thickened peritoneum [75]. Pleural effusion may be associated.

### Hemoperitoneum

Spontaneous hemoperitoneum is a very rare emergency case, which may be life-threatening [77]. Eighty percent cases of spontaneous hemoperitoneum related to endometriosis occur during pregnancy [78]. The patient usually presents with acute abdominal pain, which can be associated with signs of hemorrhagic shock.

The occurrence of hemoperitoneum in connection with endometriosis can have several origins:

- Rupture of the uterine vessels. The etiology of the condition remains poorly understood. Chronic inflammation may make the uterine vessels more friable. On the other hand, the resultant adhesions may give further tension to these vessels when the uterus is enlarged during pregnancy and during uterine contractions. A previous surgery for endometriosis may, however, weaken the vessel walls.
- Spontaneous bleeding from endometriotic implants [77].
- Rupture of an endometrioma (see above) or of a hematosalpinx.

In a series of 59 spontaneous hemoperitoneum in pregnancy, 22% and 57% were due, respectively, to the bleeding of endometriosis implants and to the erosion of utero-ovarian vessels [79]. Although primary presentation of endometriosis with spontaneous hemoperitoneum is rare, it should be kept in the differential diagnosis of hemoperitoneum in women in reproductive age. Urgent surgery is needed to identify the bleeding source and to perform selective coagulation.

## Urinary complications

Urinary tract endometriosis affects about 1–4% of patients with endometriosis and 14–20% of patients with deep infiltrating endometriosis. Bladder endometriosis is the most common (85%) of the various forms of urinary tract endometriosis, followed by endometriosis of ureter (9%), kidney (4%) and urethra (2%) [80, 81]. Urinary tract involvement may be spontaneous or secondary to pelvic surgeries, such as cesarean section or hysterectomy. Approximately 50% of patients with endometriosis of the bladder or ureter underwent pelvis surgeries in the past [82]. Acute presentation of urinary involvement by endometriosis is very rare, with very few cases of acute onset of urinary tract injury due to acute obstructive uropathy [7].

### Bladder inflammation

Endometriotic implants are often limited to the serosal surface of the bladder; however, they can also infiltrate the muscular layer and manifest as mural masses which project into the lumen [82, 83]. The posterior bladder wall, near the vesicouterine pouch, is the most frequently involved [84]. Symptomatic patients with bladder endometriosis suffer from retropubic pain or discomfort, along with irritative symptoms [83]. Cyclical hematuria is considered pathognomonic but rare, since endometriosis rarely infiltrates the mucosa deep enough to cause its ulceration. Therefore, cystoscopy may often be normal [85]. The relationship between acute abdomen and bladder endometriosis is very indirect and can manifest itself either through symptoms of acute cystitis or rarely through a bladder perforation. It has been recently shown that the risk of bladder perforation after retropubic midurethral sling placement for urinary incontinence was higher in patients with endometriosis [86].

**Fig. 9:**
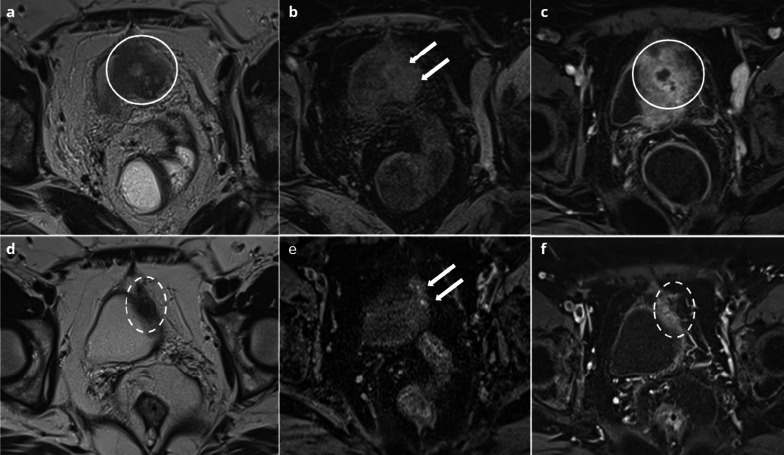
48-year-old female, with a history of severe endometriosis treated by surgery, presenting with hypogastric pain, elevated C-reactive protein (CRP) and white blood cell count. Axial T2W sequence (**a**) shows a complex cystic mass of the bladder wall (circle). **b** No hemorrhagic signal is observed on axial T1FSW sequence (arrows). After injection, axial T1FSW (**c**) depicts perivesical fat stranding and enhancement consistent with an inflammatory process (circle). Infected bladder endometriosis was suspected given the patient’s history, and a follow-up MRI was performed after antibiotherapy. Axial T2W sequence after treatment (**d**) shows a decrease in size of the mass. **e** Hyperintense spots appear on T1W FS sequences (arrows), and (**f**) inflammatory signs decrease after injection (dotted circle), consistent with the resolution of an infectious process involving the endometriotic mass

US and CT may depict a focal thickening of the bladder wall, which can be dismissed for cancer [84]. On MRI, bladder endometriosis presents as a poorly defined infiltrative or nodular lesion with low T2 signal intensity. The nodule is usually located at the level of the vesicouterine pouch within the bladder base. The presence of internal foci of high T1 and high T2 signal intensity is pathognomonic of endometriosis and rules out the diagnosis of bladder cancer [84, 87]. As observed in superinfected endometriomas, an increase in size of the nodule, along with loss of hyperintensity in T1WFS, inflammatory signs and restriction of diffusion, is suggestive of superinfection of bladder endometriosis [24] (Fig. 9).

Treatment of bladder endometriosis depends on several factors, such as age, fertility preferences, extent of disease, and severity of the symptoms. It can be medical or surgical, or a combination of the two [80].

### Urinary tract obstruction

**Fig. 10:**
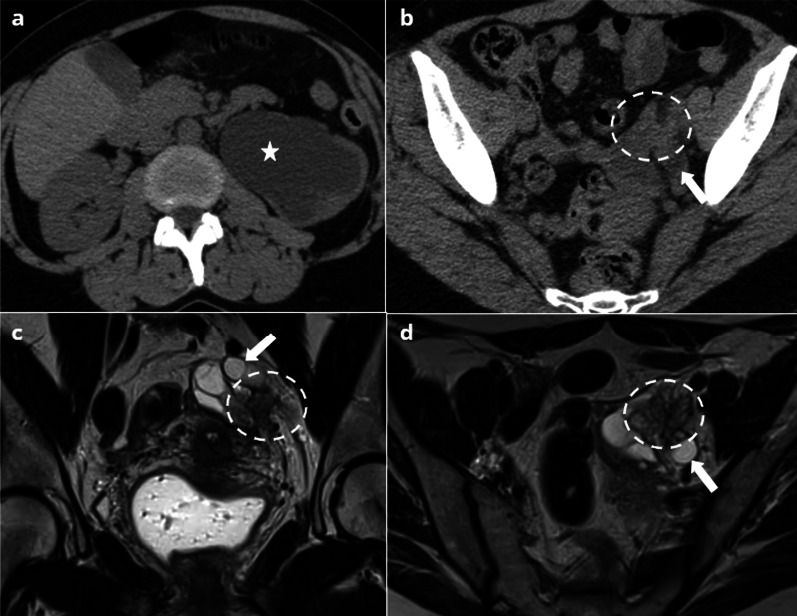
41-year-old female presenting with acute left lumbar region pain. No history of endometriosis. CT (**a**, **b**) shows a left hydronephrosis (star) and dilated ureter (arrow), up to a retractile mass located at the level of the ovarian fossa (dotted circle). T2W MRI (**c**, **d**) shows a stellar periureteral mass (dotted circle) responsible for obstruction (arrow). Nephrectomy was performed because of the complete loss of left kidney function, confirming the diagnosis of left ovarian and parametrial endometriosis, responsible for an extrinsic obstruction of the ureter

Although ureteral involvement in endometriosis is often insidious and asymptomatic (50% of cases), it may cause acute flank pain and ureteral obstruction, which require to differentiate endometriosis from urolithiasis or other obstructive processes [7]. Involvement of the ureter can be extrinsic or intrinsic with a reported ratio of 4:1 [88]. Extrinsic disease is caused by external compression of the ureter originating from adjacent disease of the ovary, broad ligaments, or uterosacral ligaments [88]. Intrinsic disease directly involves the ureteral wall, proven on histological examination [89]. US and CT may easily assess the degree of dilatation of the urinary tract, and CT may identify the location of the point of constriction. In a woman of reproductive age, a constriction point within the pelvis is highly evocative of endometriosis, especially with a clinical history of known or suspected pelvic endometriosis [90]. MRI is useful both to confirm the diagnosis, showing an irregular hypointense nodule on T2-weighted images, and to provide findings to differentiate intrinsic and extrinsic ureteral endometriosis [87] (Fig. 10).

A French study shows that if the ureter is surrounded 360° by the lesion, intrinsic involvement is confirmed in over 80% of cases, whereas if it is surrounded less than 180°, intrinsic disease is confirmed in less than 10% of cases [87]. Conversely, the identification of a soft-tissue mass within the ureter affirms intrinsic involvement in endometriosis, but may be difficult to differentiate from a urothelial lesion [91].

Distinguishing extrinsic from intrinsic implants is of importance. In both cases, surgery is recommended to protect the kidney. However, to manage extrinsic involvement, ureterolysis is often sufficient, whereas intrinsic disease will benefit from ureteral resection with reconstructive challenges depending on the length and level of ureter resected [81].

## Abdominal wall endometriosis

In patients who had abdominal or pelvic surgery (mainly gynecologic procedures, such as cesarean delivery or hysterectomy, but also non-gynecological surgery, such as appendicectomy [66]), abdominal wall endometriosis occurs within the abdominal and pelvic walls, at the site of surgical scars. However, up to 20% of cases arise spontaneously or remotely from the surgical scar [92]. The endometrial deposits may be found in subcutaneous tissues, abdominal muscles, or both [3]. Clinical signs of abdominal wall and scar endometriosis are variable. The most frequently encountered ones are cyclical localized pain and/or swelling, worsening of the symptoms during the first phase of the menstrual cycle [92]. However, in some cases, the patient may present with clinical findings of acute abdomen. In general, outside the specific context of endometriosis acute abdominal pain is actually considered of parietal origin in up to 10% of patients. Therefore, abdominal wall tenderness may be looked for in the clinical evaluation of acute abdomen [93].

**Fig. 11:**
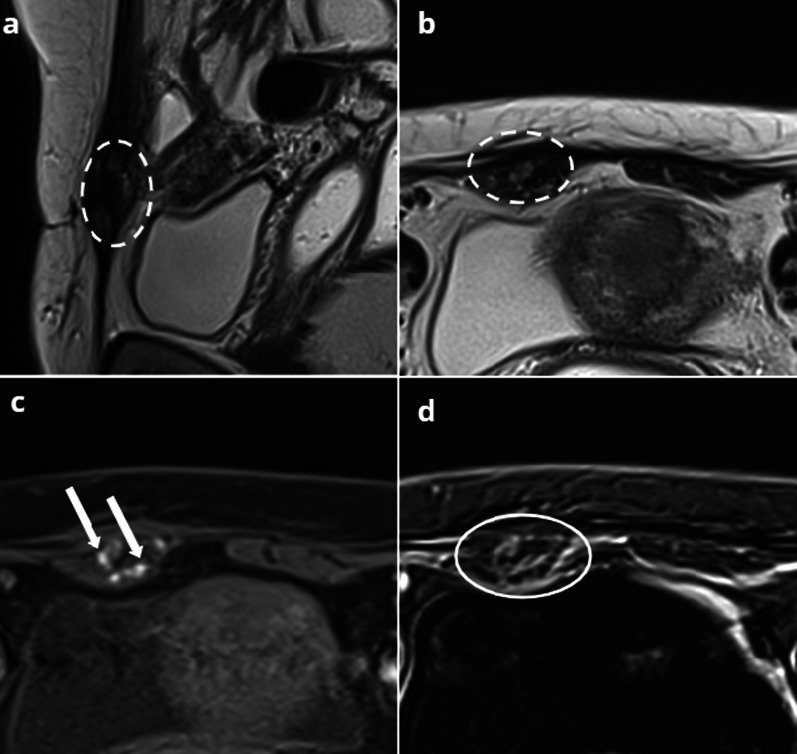
41-year-old female presenting with hypogastric anterior parietal pain and history of C-section. T2W sequences show an irregular mass located within the right rectus muscle, on the C-section scar (dotted circle) (**a**, **b**). Axial T1W FS sequence shows hemorrhagic hyperintense spots within the nodule (arrows) (**c**). After contrast injection, on subtraction sequences, the mass shows heterogeneous enhancement (plain circle) (**d**)

US is the first-line imaging modality in a context of suspicion of parietal wall endometriosis. It shows a hypoechoic irregular nodule or mass, sometimes containing small cystic areas [3]. CT shows an infiltrating soft tissue enhancing mass within a prior abdominal scar [13], but it is marginally helpful to characterize abdominal wall endometriosis or evaluate its extent. MRI is more specific for the diagnosis, especially when small hemorrhagic areas in high T1 signal are present within the fibrous soft-tissue mass [24] (Fig. 11). Moreover, MRI provides a complete anatomic delineation of the disease [94] and, in particular, the precise localization of the lesion relative to the aponeurosis (anterior or posterior) of the rectus muscle, which is helpful in planning surgical management. Of note, anterior saturation bands should be displaced when suspecting wall endometriosis. Differential diagnoses mainly include desmoid tumors and intra-muscular hematomas. Desmoid tumors may also arise from parietal wall scars, and tend to occur during pregnancy, but usually show iso- to hyperintense signal on T2-weighted sequences and isointense signal on T1-weighted sequences. Hematomas of the rectus muscle show high signal on T1-weighted sequences, but clinical context is usually suggestive of the diagnosis (trauma, anticoagulant medication) [95].

Because of the overlapping with other abdominal parietal masses, ultrasound-guided biopsy is theoretically recommended, although sometimes not performed, prior to surgical or percutaneous ablation (High-Intensity Focused Ultrasound therapy or cryoablation) [96]. However, in our practice, when endometriosis is known and MRI shows hypersignal of glandular endometrial cyst on T1-weighted sequence, abdominal wall endometriosis is managed without the need of diagnostic biopsy.

## Diagnostic strategy

The diagnosis of acute abdominal or pelvic pain related to endometriosis is difficult for several reasons: these complications are rare; they can be dismissed as dysmenorrhea in patients with known endometriosis or can reveal a previously unknown endometriosis. However, acute events in women with endometriosis can represent life-threating conditions that require emergent medical treatment and, more often, surgical management. Therefore, we have collected useful data to provide guidance when evoking a complication of endometriosis in a patient with a clinical picture of acute abdomen:

1. Integrate a context consistent with endometriosis, if endometriosis is unknown:
  Women of childbearing age;
  History of chronic pelvic pain or symptoms such as intermenstrual bleeding, dysmenorrhea, dyspareunia, dyschezia or dysuria;
  Infertility of unknown cause;
  Previous imaging showing a complex ovarian mass or a hydrosalpinx.
2. Analyze the type of pain, look for an epidemiological context and imaging signs which can be integrated in a context of acute complications of endometriosis (Table 2).
3. Look for other imaging features suggestive of deep endometriosis:
  Pelvic hemorrhagic or fibrotic implants;
  Endometriomas;
  Kissing ovaries.

**Table 2:** Keys to evoke the diagnosis of complicated endometriosis

Clinical finding	Cause	Warning context	Modality	Imaging features	Differential diagnosis
Acute pelvic pain	PID	ART	US/MRI	/	/
	Endometrioma surinfection	ART	US/MRI	Loss of hypersignal T1, loss of shading sign, very low ADC value	Tubo-ovarian abscess
	Endometrioma rupture	/	US/CT	Ascite, hyperintense on T1, distorted endometrioma	Ruptured corpus luteal cyst
Right lower quadrant pain	Endometriotic appendicitis	/	US/CT	Soft tissue mass within appendix/Mucocele	Appendicitis/ Appendix tumor
Renal colic	Ureteral obstruction by endometriosis	/	US/CT	Extrinsic compression of the distal ureter by a retractile mass	Retroperitoneal fibrosis/urothelial lesion
Parietal pain	Abdominal wall endometriosis	Cesarean delivery/pelvic surgery	US/MRI	Enhancing wall mass with irregular margin, and cystic components in hypersignal T1/T2	Desmoid tumor
Bowel obstruction	LBO	/	CT/MRI	Extrinsic soft-tissue mass compressing the sigmoid colon, asymmetric thickening of the colonic wall	Colon cancer
	SBO	/	CT	Non-specific	/
	Intussusception	/	CT	Non-specific	/
Peritoneal syndrome	Spontaneous hemoperitoneum	Pregnancy	US/CT/MRI	Ascite, hyperintense on T1 (± distorted endometrioma)	/
	Inflammatory peritoneal	/	CT	Non-specific	/
	Bowel perforation	/	CT	Non-specific	/

*PID* pelvic Inflammatory disease, *ART* assisted reproductive therapy, *US* ultrasonography, *MRI* magnetic resonance imaging, *ADC* apparent coefficient diffusion, *CT* computed tomography, *LBO* large bowel obstruction, *SBO* small bowel obstruction

## Conclusion

Endometriosis is a common disease, which can present with a variety of symptoms, and not exclusively chronic symptoms. A complication of endometriosis should always be kept in mind as a possible cause for acute symptoms in a woman of reproductive age, and the patient’s history should always be asked for and considered. MRI is the best imaging modality to reach the diagnosis of endometriosis, but an accurate diagnosis is possible on CT, especially in the presence of stellar, mildly enhanced, infiltrative lesions in suggestive areas. Save the complications of endometriomas, the main differential diagnoses are malignant tumors.

## Data Availability

The datasets used and/or analyzed during the current study are available from the corresponding author on reasonable request.

## Abbreviations

ADC: Apparent coefficient diffusion
ART: Assisted reproductive therapy
CRP: C-reactive protein
CT: Computed tomography
HCG: Human chorionic gonadotropin
LBO: Large bowel obstruction
MRI: Magnetic resonance imaging
PID: Pelvic inflammatory disease
SBO: Small bowel obstruction
US: Ultrasonography
